# Resuscitative Endovascular Balloon Occlusion of the Aorta (REBOA): in action or on the shelf? A comprehensive analysis of current practices in Germany, Austria, and Switzerland - insights from the Traumaregister DGU^®^

**DOI:** 10.1186/s12873-025-01417-3

**Published:** 2025-11-22

**Authors:** Jan Stein, Oliver Cruciger, Christopher Ull, Aileen Spieckermann, Rolf Lefering, Thomas Armin Schildhauer, Uwe Hamsen, the TraumaRegister DGU

**Affiliations:** 1 BG University Hospital Bergmannsheil, Ruhr University Bochum, Bürkle De La Camp Platz 1, 44789 Bochum, Germany; 2 Institute for Research in Operative Medicine (IFOM), University Witten/Herdecke, Witten/Herdecke, Germany; 3 Committee on Emergency Medicine, Intensive Care and Trauma Management (Sektion NIS) of the German Trauma Society (DGU), Munich, Germany

**Keywords:** REBOA, Trauma management, Haemorrhagic shock, Emergency interventions, Haemorrhage control, TraumaRegister DGU®, Trauma epidemiology

## Abstract

**Purpose:**

The use of Resuscitative Endovascular Balloon Occlusion of the Aorta (REBOA) for temporary bleeding control in severely injured patients remains controversial. Epidemiological data from Germany, Austria and Switzerland are lacking. The objective of this study was therefore to collect data on the use of REBOA intervention and the characteristics of the affected patient population.

**Methods:**

A retrospective analysis of the TraumaRegister DGU^®^ between January 2020 and December 2022 was conducted to evaluate the frequency of REBOA interventions, injury patterns, injury severity, epidemiology and additional therapies received by patients treated with REBOA.

**Results:**

Between 2020 and 2022, 95,510 patients were documented in Germany, Austria and Switzerland (DACH), of whom 62 received REBOA. 44 of 62 patients (71%) were under 60 years of age and 57 (92%) suffered a blunt trauma. Severe injury (AIS ≥ 3) was present in the following regions: head 36%, thorax 77%, abdomen 58%, extremities 65%. 23 of 59 patients (39%) did not have a systolic blood pressure ≤ 90 mmHg. Of the 62 patients, 42 (68%) received at least one unit of packed red blood cells (PRBC), and 24 (39%) received ≥ 10 PRBCs within the first 48 h. Thoracotomy was performed in nine patients (15%), laparotomy in 24 (39%), and 15 (23%) underwent surgical pelvic stabilisation. The expected mortality according to the RISC II score was 43%, while the observed mortality was 45%. Fourteen REBOAs (22%) were performed at one centre; two centres conducted six (10%) and seven (11%) procedures, and 27 centres performed a single REBOA within the three-year period.

**Conclusion:**

The use of REBOA is extremely rare in the DACH. Only a few centres perform REBOA more than once per year. A striking proportion of patients treated with REBOA had no hypotension, received no blood or massive transfusions, and underwent no emergency surgery, which may indicate that some patients were not in severe haemorrhage when REBOA was applied. On average, the REBOA-treated cohort was severely injured and critically ill. Whether REBOA use was beneficial and/or necessary in these patients cannot be determined from the present study.

## Introduction

Despite significant advances in the medical management of polytrauma patients, one issue that initially appears manageable and treatable remains a leading cause of mortality: uncontrolled haemorrhage in patients suffering from massive haemorrhagic shock [1]. As part of the multimodal approach to the treatment of traumatic haemorrhagic shock, various conservative strategies — including cardiovascular support therapy, patient blood management, and coagulation management — alongside surgical interventions such as damage control surgery, have been established [2]. In cases of severe refractory haemorrhagic shock, discussions often turn to highly invasive procedures such as resuscitative thoracotomy [3]. Recently, a less invasive intervention that offers promising haemorrhage control has gained increasing attention: Resuscitative Endovascular Balloon Occlusion of the Aorta (REBOA) [4–6]. The main aim of these interventions is temporary bleeding control until definitive haemostasis can be achieved.

To date, no conclusive evidence supporting the use of REBOA has been obtained from published case analyses, which have primarily been conducted in Japan and the United States [7, 8]. Current data suggest that REBOA may be a viable treatment option for patients experiencing haemorrhagic shock that is refractory to conventional treatment approaches and in whom non-compressible bleeding in the thoracic, abdominal or pelvic regions is suspected [9]. However, a recent randomised controlled trial was prematurely terminated due to a prespecified stopping rule for harm observed in the intervention group receiving REBOA [10].

There is currently a lack of data regarding the epidemiology and current practice of REBOA use in comparable pre-hospital and in-hospital trauma care settings in Germany, Austria and Switzerland (DACH). Therefore, this study was undertaken to address this gap in knowledge.

## Materials and methods

### Study design

A registry evaluation of the TraumaRegister DGU^®^ of the German Trauma Society (Deutsche Gesellschaft für Unfallchirurgie, TR-DGU^®^) was conducted for the period from January 2020 to December 2022.

The TR-DGU^®^ was established in 1993 with the objective of providing pseudonymised and standardised documentation of severely injured patients across multiple centres. Data are prospectively collected in four consecutive phases, from the site of the accident to hospital discharge: (a) pre-hospital phase, (b) emergency room and initial surgery, (c) intensive care unit (ICU), and (d) discharge. The registry includes comprehensive data on patient demographics, injury patterns, comorbidities, pre- and in-hospital management, ICU course, relevant laboratory findings (including transfusion data), and patient outcomes. Inclusion criteria encompass admission to hospital via the emergency room with subsequent ICU/intensive care monitoring (ICM), or patients who arrive at hospital with vital signs and die prior to ICU admission.

The infrastructure for data documentation, management, and analysis is provided by the AUC – Academy for Trauma Surgery (AUC – Akademie der Unfallchirurgie GmbH), an entity affiliated with the German Trauma Society. Scientific oversight is maintained by the Committee on Emergency Medicine, Intensive Care, and Trauma Management (Section NIS) of the German Trauma Society. Participating hospitals submit pseudonymised data into a central database via a web-based application [11]. Scientific data analysis follows a peer review process in accordance with the publication guidelines of the TR-DGU^®^. This study is registered as TR-DGU^®^ project ID 2023-037.

Although the majority of participating hospitals are located in Germany (90%), an increasing number of institutions from other countries — including Austria, Belgium, China, Finland, Luxembourg, Slovenia, Switzerland, the Netherlands, and the United Arab Emirates — also contribute data. Currently, more than 38,000 cases from approximately 700 hospitals are entered into the database annually. Participation in the TR-DGU^®^ is voluntary; however, for hospitals associated with the TraumaNetzwerk DGU^®^, entry of at least a basic dataset is mandatory for quality assurance purposes.

Since the 2020 dataset revision, the use of REBOA has been included as one of eleven documented emergency interventions/operations. In addition, ‘balloon occlusion’ has also been available as a type of surgery for specific injuries since 2020. Both options were used to identify the patient group. One case was excluded after individual case evaluation owing to incorrect documentation. All remaining cases of REBOA usage in DACH were included in this analysis.

### Ethical approval/ trial registration

The study was approved by the Ethics Committee of Westfalen-Lippe (ethics committee of the regional medical association (Ärztekammer Westfalen-Lippe), reference number 2024-312-f-S). It adhered to the ethical standards set forth in the 1964 Declaration of Helsinki and its subsequent amendments. As a retrospective study, it was not registered with ClinicalTrials.gov or any other trial registry. Therefore, a clinical trial number is not applicable.

### Consent for participation and publication

Informed consent for inclusion in the TR-DGU^®^ is obtained centrally by participating hospitals at the time of registry documentation in accordance with national data protection and ethical standards. Consequently, only patients (or, where applicable, their legal representatives) who provided consent for data entry and scientific use are included in the registry. In accordance with Article 1(1) of the General Data Protection Regulation, basic data protection does not apply to the personal data of dead persons. Therefore, dead patients were included in the study without explicit consent [12].

### Funding

Open Access funding enabled and organized by Projekt DEAL in cooperation with Ruhr University Bochum, Germany.

### Statistics

Statistical analyses were performed using SPSS Statistics version 29 (IBM Corporation, Armonk, NY, USA). Data visualisations were created using Microsoft^®^ Excel (version 16.84) and Microsoft^®^ PowerPoint (version 16.82).

## Results

### Patients characteristics

Between 01/2020 and 12/2022, a total of 95,510 cases were documented in the DACH region, of which 62 patients (0.065%) received REBOA. Table 1 provides an overview of patient characteristics, injury patterns, and administered therapies. REBOA was utilised across all age groups, ranging from 13 to 90 years (mean age 48). The predominant trauma mechanism was blunt trauma, accounting for 92% of cases, while penetrating trauma was reported in only 8%. The thorax was the most frequently affected body region (77% of cases), followed by extremity injuries (65%).

Regarding injury severity, the distribution of the maximum Abbreviated Injury Scale (AIS) score per patient indicated that all patients had at least one AIS score of 3 or higher. The most frequently recorded maximum AIS score was 5, observed in 56% of patients. The mean Injury Severity Score (ISS) was 43 (SD 20), with 89% of patients having an ISS ≥ 16. The mean base excess (BE) on admission was − 12 (SD 7), and 68% of patients presented with acidosis (BE ≤ − 6).

**Fig. 1:**
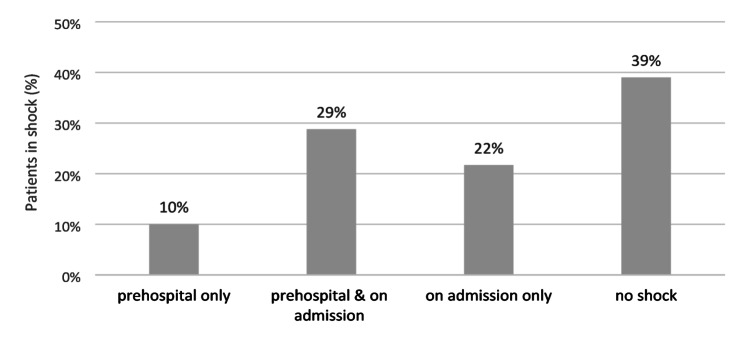
Percentage of patients with documented shock, defined as decrease of systolic blood pressure ≤90mmHg. In 3 cases, no blood pressure values are documented

A shock event, defined as a systolic blood pressure of ≤ 90 mmHg in according to trauma guidelines [13], was documented in 36 cases (Fig. 1). In 22% (13/59) of cases, shock developed only after hospital admission. In 29% (17/59) of cases, shock occurred prehospital and persisted upon hospital arrival. In the remaining 10% (6/59), shock occurred prehospital but had resolved by the time of hospital admission. No shock event was observed in 23 of 59 patients (39%). In three cases, no blood pressure values were documented. In some instances, blood pressure was recorded only prehospital or on admission.

Endotracheal intubation was performed in 56% of cases (35/62), whereas alternative airway management methods were employed in 3% (2/62). Volume resuscitation was administered to 82% of patients (51/62). Circulatory support, including the administration of catecholamines, was provided to 37% (23/62). Cardiac arrest occurred in 15% of cases (9/62), necessitating cardiopulmonary resuscitation (CPR). The majority of patients (68%, 42/62) received at least one unit of packed red blood cells (PRBCs), while mass transfusion, defined as the administration of more than 10 units of PRBCs, was required in 39% (24/62). Thoracotomy was performed in 15% (9/62) of patients, and laparotomy was conducted in 39% (24/62) to access the abdominal cavity.

**Table 1:** Patients characteristics, injury pattern and received therapy

**Age distribution (in years)**	
< 60 n (%)	44 (71)
60–69 n (%)	8 (13)
70–79 n (%)	5 (8)
80 + n (%)	5 (8)
Mean age (SD)	48 (20)
**ASA**	
ASA 1/2 n (%)	42 (81)
ASA3/4 n (%)	10 (19)
**Type of accident**	
Car n (%)	13 (22)
Motorcycle n (%)	19 (32)
Bike n (%)	4 (7)
Pedestrian n (%)	2 (3)
High fall (> 3 m) n (%)	8 (13)
Low fall (< 3 m) n (%)	6 (10)
Other n (%)	7 (12)
**Type of trauma**	
Blunt trauma n (%)	57 (92)
Penetrating trauma n (%)	5 (8)
**AIS ≥ 3**	
Head n (%)	22 (36)
Thorax n (%)	48 (77)
Abdomen n (%)	36 (58)
Extremities n (%)	40 (65)
**Maximum AIS**	
3 n (%)	11 (18)
4 n (%)	13 (21)
5 n (%)	35 (56)
6 n (%)	3 (5)
**ISS Score**	
≥ 16 n (%)	55 (89)
Mean ISS (SD)	43 (20)
**Base excess**	
BE ≤ -6 n	39 (68)
Mean BE (SD)	-12 (7)
**Pre-hospital Therapy**	
Intubation n (%)	35 (56)
Alternative airway n (%)	2 (3)
Volume received n (%)	51 (82)
Catecholamines n (%)	23 (37)
CPR n (%)	9 (15)
Pelvic belt n (%)	30 (48)
Tranexamic acid n (%)	25 (40)
**In-hospital Therapy**	
PRBC transfusion n (%)	42 (68)
≥10 PRBC transfusion n (%)	24 (39)
Emergency intervention n (%)	56 (90)
Thoracotomy n (%)	9 (15)
Laparotomy n (%)	24 (39)
Pelvic stabilisation n (%)	18 (29)

ASA: American Society of Anaesthesiologists. AIS: Abbreviated Injury Scale. ISS: Injury Severity Score. Shock: decrease of systolic blood pressure ≤90mmHg. PRBC: packed red blood cells. CPR: cardiopulmonary resuscitation. Pelvic stabilization: c-clamp treatment and/or supraacetabular external fixation

**Fig. 2:**
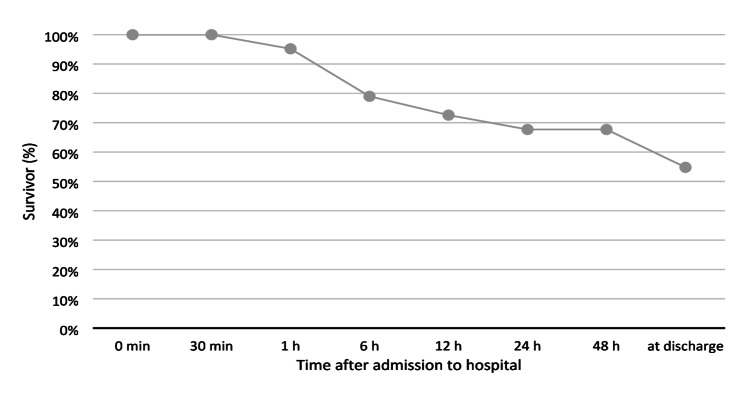
Percentage of surviving patients correlated to time after hospital admission

The survival rates of patients who underwent REBOA intervention are presented in Fig. 2. No deaths were recorded within the first 30 min following hospital admission. However, within the first hour, three patients died. Over the next five hours, additional 10 patients succumbed to their injuries. Four more patients died within 12 h, and three additional deaths occurred by 24 h post-admission. By the end of the hospital stay, eight further deaths had occurred, resulting in an overall survival rate of approximately 55%. The mortality rate at 24 h post-admission was 32%, with a total mortality rate of 45% for the entire hospital stay (*n* = 28). The predicted mortality rate, based on the RISC II score (Revised Injury Severity Classification, version II [14]), was 43% for the study cohort.

### Distribution of hospitals using REBOA

**Fig. 3:**
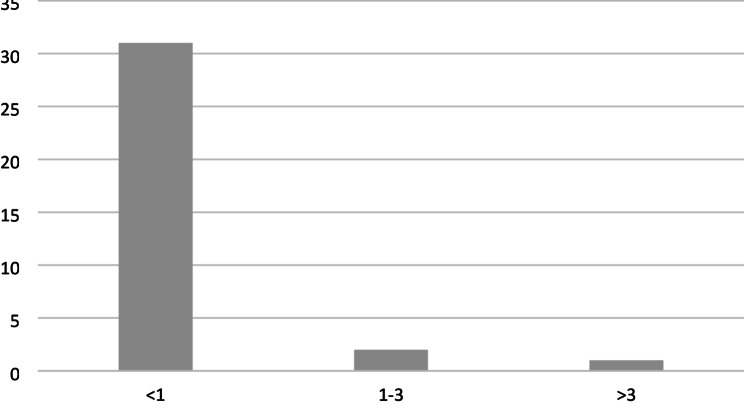
Number of hospitals performing < 1, 1–3 and > 3 REBOA intervention per year

Figure 3 illustrates the distribution of implanted REBOA catheters across participating hospitals (*n* = 34). Among the documented REBOA interventions, 14 (23%) were performed at a single hospital. Additional 7 (11%) and 6 (10%) interventions were conducted at two other hospitals. The remaining 35 documented REBOA procedures were distributed among 31 hospitals. Notably, 27 hospitals each implanted only one REBOA catheter during the study period.

## Discussion

This study demonstrates that the utilisation of REBOA is infrequent in DACH region. To the best of our knowledge, this research is the first to delineate the current epidemiology of REBOA within these healthcare systems in trauma care. Among the 95,510 documented cases, REBOA was performed in 62 instances, corresponding to a rate of 0.065%. These 62 interventions were conducted across only 34 trauma centres, with just three centres performing more than one REBOA procedure annually.

Gorman et al. [15] emphasised the significance of experience in both the indication for and execution of the REBOA procedure. Their analysis of the AORTA registry revealed a correlation between procedural volume and improved patient outcomes.

The UK-REBOA trial, the only recent randomised controlled trial, compared standard care alone to REBOA as an adjunct to standard care [10]. The authors concluded that REBOA may be associated with increased mortality, as evidenced by 54% of patients in the REBOA group and 42% in the control group having died by 90 days post-treatment (Odds Ratio 1.58). This study was conducted across 16 major trauma centres in the UK, enrolling 90 patients, of whom 46 were randomly assigned to receive REBOA. Over a 4.5-year enrolment period, only four centres were able to enrol more than one patient per year. The authors identified the low institutional case volume of exsanguinated patients relative to other countries as a limitation of their study, attributing this to high road safety standards and low levels of interpersonal violence in the UK. These findings align with those of our study and other analyses regarding early mortality due to haemorrhage in the TR-DGU^®^ [16].

While the UK-REBOA trial was halted due to potential harm associated with the REBOA intervention, several other registry-based studies suggest that REBOA may confer survival benefits when applied to appropriately selected patients [17]. In particular, compared with thoracotomy involving aortic cross-clamping, a review study demonstrated a survival advantage associated with REBOA therapy [17]. Our present study could not assess the impact of REBOA on survival for the 62 cases due to the limited number of cases and the absence of critical detailed parameters. Nevertheless, we observed that the overall mortality rate (45%) was nearly identical to the mortality rate predicted by the RISC II score (43%) [14], which is notably similar to the mortality rates reported in the UK-REBOA trial (54% for REBOA plus standard care vs. 42% for standard care alone) [10].

The indication for REBOA is both complex and critical. Patients may either be untreatably deteriorated or may not require REBOA as a bridge to definitive bleeding control if the extent of bleeding is overestimated. In the UK-REBOA trial [10], of the 46 patients assigned to REBOA, 17 were not treated with the intervention due to prior stabilisation, while two were excluded due to deterioration. The inclusion criteria specified an age greater than 15 years and confirmed or suspected life-threatening torso haemorrhage deemed amenable to adjunctive REBOA treatment. Patients with known or suspected pregnancy or with injuries clearly deemed non-survivable were excluded. In our study, the parameters leading to REBOA remain speculative. The parameter defined as “shock” in the registry, characterised by a systolic blood pressure of equal to or less than 90 mmHg, occurred in 36 cases (61%). Conversely, 23 cases (39%) exhibited neither pre-hospital nor in-hospital signs of shock. Precise data on individual indications for REBOA intervention are missing due to pseudonymised registry analysis. Therefore, it remains unclear whether haemodynamic deterioration occurred in the remaining 23 cases without documented shock on initial assessment or on admission as an indication for REBOA.

Surprisingly, the administration of REBOA was not consistently accompanied by adjunctive measures typically used in the management of severe haemorrhagic shock, such as massive transfusion, administration of tranexamic acid, or circulatory support therapy. Only 39% of patients received a massive transfusion (defined as ≥ 10 units of PRBCs), 68% of patients received only a single unit of PRBCs. Furthermore, merely 40% were administered tranexamic acid in the pre-hospital setting, and only 37% received catecholamines for circulatory support.

This apparent lack of correlation between REBOA use and established shock management protocols raises questions regarding the underlying indication for its application. It can only be partially explained by the fact that a small number of patients (*n* = 3) succumbed within the first hour after admission, before further resuscitative measures could be initiated. Alternatively, it is conceivable that in some cases REBOA was used as a pre-emptive or exploratory measure rather than as part of a comprehensive haemostatic resuscitation strategy. This observation may reflect variability in institutional experience, clinical judgement, or local protocols regarding the use of REBOA in trauma care.

Regarding injury severity, 11 patients had a maximum AIS score of 3. In contrast, within the study cohort of Jansen et al. in the UK-REBOA trial, only 3 out of 90 patients (3%) had an ISS of less than 16, and 52% of all patients presented with a systolic blood pressure of less than 90 mmHg in the ED. These findings may suggest an overestimation of the actual bleeding severity in some patients, placing them at high risk for complications arising from REBOA.

The complication rate associated with REBOA implantation can be as high as 54% [18], with serious complications including pseudoaneurysms, dissections, local infections, distal embolisms leading to ischaemia, compartment syndromes, amputations, ischaemia-reperfusion syndromes, spinal cord injuries, acute kidney injury, and secondary acute respiratory distress syndrome [19–21]. Several studies are currently being conducted to evaluate complication rates more precisely, including in terms of complete vs. incomplete occlusion of the aorta [22–24]. Notably, no complications were reported in the UK-REBOA trial, although the lack of high-volume experience among participating trauma centres was a significant limitation of that study. In the current investigation, we were unable to report on complication rates, as no data regarding complications are collected within the registry. Furthermore, we could not ascertain the training experience of trauma centre personnel with REBOA or the time elapsed from admission to REBOA insertion. Additionally, while we can assume that REBOA was primarily inserted in the ED, some REBOA catheters may have been placed pre-hospital, adding uncertainty to our parameter descriptions.

In DACH, there are currently no established nationwide standards for the indication or availability of REBOA in the pre-hospital setting. The updated German guideline for the management of major trauma, released in 2022, recommends that REBOA may be employed in cases of traumatic cardiac arrest for temporary proximal bleeding control (Good Practice Point) and states that “in patients with severe haemorrhagic shock due to non-compressible bleeding of the torso below the diaphragm, REBOA might be utilised until definitive bleeding control is achieved” (grade of evidence 0) [25].

In our study, we identified nine out of 62 patients with cardiac arrest in the ED who received REBOA, but we cannot determine whether REBOA was performed before or after the onset of cardiac arrest. The German Resuscitation Council recommends REBOA as a potential option for temporary bleeding control, contingent upon appropriate “expertise, equipment, environment, and elapsed time” [26].

### Limitations

This study has several limitations that warrant consideration. The registry-based nature of our data lacks comprehensive details regarding the clinical course of patients during treatment in the emergency department, including vital signs recorded before and after REBOA implantation, as well as the induction time and timing of REBOA insertion. Additionally, the number of patients who were potential candidates for REBOA but were either deemed too stable or too unstable prior to implementation, as well as those in whom REBOA was planned but found to be technically unfeasible, remains unknown. In cases of pre-hospital REBOA usage, it is uncertain whether these procedures were accurately documented as emergency department interventions. Another limitation is that the TR-DGU^®^ does not collect data on the training level or experience of the treating providers. This appears to be a relevant factor, particularly given that REBOA interventions are rarely performed and are associated with high peri-procedural complication rates.

Due to these limitations in detail, the present study cannot contribute to an evaluation of the benefits versus harms associated with REBOA. Nevertheless, the registry-based design provides valuable insights into the incidence and epidemiology of REBOA utilisation in the DACH region.

## Conclusions

At present, the use of REBOA is not established as a standard practice in trauma management within the DACH region. Owing to the rarity of cases, the TR-DGU^®^ does not provide sufficient data to evaluate the potential benefits or harms associated with REBOA application. Furthermore, in several cases within our cohort, the indication for REBOA use appeared unclear, as subsequent treatment consistent with the management of severe haemorrhagic shock—such as mass transfusion, volume replacement, or catecholamine therapy—was not initiated. This discrepancy may indicate that REBOA was deployed in patients who were not in profound shock at the time of intervention. The indications for REBOA therefore remain complex, and trauma teams may be at risk of overestimating the extent of blood loss and injury severity, leading to potentially inappropriate use in selected cases.

## Data Availability

The data used in this study were obtained from the TR-DGU^®^ of the German Trauma Society. Access to the data is restricted to maintain patient confidentiality. Data can be made available from the corresponding author upon reasonable request and with permission from the TR-DGU^®^.

## Abbreviations

AIS: Abbreviated Injury Scale
ASA: American Society of Anaesthesiologists
BE: Base excess
CPR: Cardiopulmonary resuscitation
DACH: Germany, Austria and Switzerland
ED: Emergency department
ICM: Intensive care monitoring
ICU: Intensive care unit
ISS: Injury Severity Score
PRBC: Packed red blood cells
REBOA: Resuscitative Endovascular Balloon Occlusion of the Aorta
RISC II: Revised Injury Severity Classification (version II)
SD: Standard deviation
TR-DGU®: Traumaregister^®^ der deutschen Gesellschaft für Unfallchirurgie
UK: United Kingdom
